# Non-Thermal Atmospheric Pressure Plasma Inhibits Thyroid Papillary Cancer Cell Invasion via Cytoskeletal Modulation, Altered MMP-2/-9/uPA Activity

**DOI:** 10.1371/journal.pone.0092198

**Published:** 2014-03-25

**Authors:** Jae Won Chang, Sung Un Kang, Yoo Seob Shin, Kang Il Kim, Seong Jin Seo, Sang Sik Yang, Jong-Soo Lee, Eunpyo Moon, Keunho Lee, Chul-Ho Kim

**Affiliations:** 1 Department of Otolaryngology, School of Medicine, Ajou University, Suwon, Korea; 2 Department of Electrical and Computer Engineering, Ajou University, Suwon, Korea; 3 Department of Molecular Science and Technology and Department of Life Science, Ajou University, Suwon, Korea; 4 Plasma Systems and Materials (PSM) America Inc., Colorado Springs, Colorado, United States of America; Stony Brook University, United States of America

## Abstract

Plasma, the fourth state of matter, is defined as a partially or completely ionized gas that includes a mixture of electrons and ions. Advances in plasma physics have made it possible to use non-thermal atmospheric pressure plasma (NTP) in cancer research. However, previous studies have focused mainly on apoptotic cancer cell death mediated by NTP as a potential cancer therapy. In this study, we investigated the effect of NTP on invasion or metastasis, as well as the mechanism by which plasma induces anti-migration and anti-invasion properties in human thyroid papillary cancer cell lines (BHP10-3 and TPC1). Wound healing, pull-down, and Transwell assays demonstrated that NTP reduced cell migration and invasion. In addition, NTP induced morphological changes and cytoskeletal rearrangements, as detected by scanning electron microscopy and immunocytochemistry. We also examined matrix metalloproteinase (MMP)-2/-9 and urokinase-type plasminogen activator (uPA) activity using gelatin zymography, uPA assays and RT-PCR. FAK, Src, and paxillin expression was detected using Western blot analyses and immunocytochemistry. NTP decreased FAK, Src, and paxillin expression as well as MMP/uPA activity. In conclusion, NTP inhibited the invasion and metastasis of BHP10-3 and TPC1 cells by decreasing MMP-2/-9 and uPA activities and rearranging the cytoskeleton, which is regulated by the FAK/Src complex. These findings suggest novel actions for NTP and may aid in the development of new therapeutic strategies for locally invasive and metastatic cancers.

## Introduction

Thyroid papillary carcinoma is one of the most common malignancies worldwide and generally shows indolent character [1]. However, it can sometimes be aggressive, with extracapsular spread, strap muscle, recurrent laryngeal nerve, and tracheal invasion, as well as metastasis to lymph nodes. In rare cases, thyroid papillary cancer can metastasize to lung or bone [2], [3]. The presence of local or distant metastases affects tumor recurrence, patient survival rates, and quality of life, thereby leading to poor prognoses [4]. Therefore, it is necessary to discover novel ways to prevent the aggressive features of invasion and metastasis in thyroid papillary cancer.

Plasma medicine is a rapidly growing field involving a novel treatment modality [5], [6]. Different plasma sources and plasma devices are used for several indications, including disinfection [7], wound healing [8], blood coagulation [9], and cancer cell death [10]. Moreover, technological advances have permitted the generation of plasmas at room temperature and atmospheric pressure (non-thermal atmospheric pressure plasma, NTP) [10]–[12]. NTPs have been reported to have anti-cancer activities in various tissues, including lung cancer [5], colorectal cancer [10]–[12], and melanoma [13], suggesting a new anti-tumor therapeutic modality. In addition, our group previously showed that NTP induced a growth arrest and retarded tumor invasion in colorectal cancer cells [10], [11]. However, the anti-cancer mechanism of NTP has mainly focused on apoptosis-related mechanisms with increased reactive oxygen species or redox imbalances [14].

Tumor invasion to surrounding tissues or metastasis to distant organs is the predominant cause of mortality in patients with cancer [15]. Therefore, emphasis has been placed on inhibiting cancer invasion and metastasis. Many lines of evidence demonstrate that a complex process of interdependent steps, including cell migration, invasion, surface adhesion, and degradation of the extracellular matrix (ECM), is closely related with tumor invasion and/or metastasis and is regulated by extremely complicated mechanisms [16]. Previously, we suggested that NTP treatment decreased cell migration and invasion in colorectal cancer cells [10]. However, the mechanisms involved in the plasma-induced inhibition of cell migration and invasion are not fully understood.

The purpose of this study was to explore the inhibitory effects of NTP on the migration and invasion of human thyroid cancer cell lines. To the best of our knowledge, this is the first study evaluating the anti-cancer effect of NTP on cell migration and invasion associated with cytoskeletal modulation and changes of matrix metalloproteinase (MMP)-2/-9/urokinase-type plasminogen activator (uPA) activities.

## Materials and Methods

### Cell lines and reagents

The human thyroid papillary carcinoma cell lines BHP10-3 and TPC1 were purchased from the American Type Culture Collection (Manassas, VA, USA). The normal thyroid cell line Nthy-ori 3-1 was kindly presented by prof. Minho Song (Chungnam National University, Korea). BHP10-3 and Nthy-ori 3-1 cells were maintained in RPMI 1640 medium (Gibco, Carlsbad, CA, USA), while TPC1 cells were maintained in Dulbecco's modified Eagle's medium/Ham's nutrient mixture F-12 (DMEM/F12; Gibco), supplemented with 10% fetal bovine serum (FBS) and 100 U/ml penicillin-streptomycin (Gibco). The cells were maintained at 37°C with 5% CO_2_ under humidified conditions.

### Experimental system specifications

As described previously [11], we designed and manufactured a spray-type NTP system with a newly designed arc-free and antistatic plate that provides a uniform plasma jet for biomedical research applications. This system shows high efficiency for the surface modification of bio-samples at low temperatures, which is critical for biological experiments.

**Figure 1:**
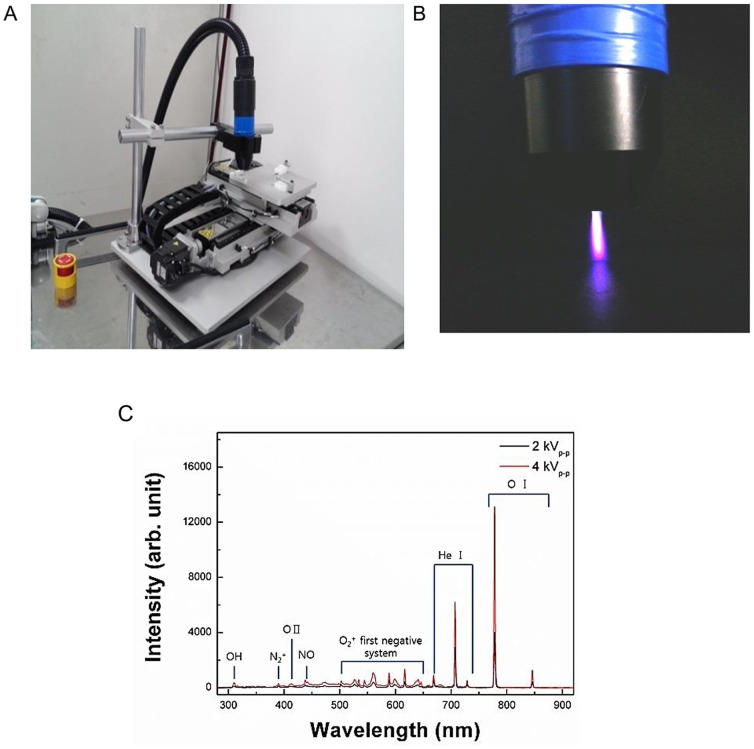
Non-thermal atmospheric pressure plasma (NTP) generating system used in this study (A) Photograph of our spray-type plasma generating system. (B) Optical emission spectra of the He and O_2_ gas mixture plasma according to the electric intensity (2 or 4 kV) in the range of 280–920 nm. (C) Image of the “torch with spray-type” plasma jet with He and O_2_. The visible plasma had a length of approximately 2.5 cm that varied with the gas flow and voltage.

The technical specifications of the plasma system (“torch with spray-type”) are shown in Figs. 1A and B. The specifications of the power supply for this system are 2 kV minimum, 13 kV maximum, and a mean frequency of 20–30 kHz; these specifications can vary with the type and amount of gas used. In this study, helium (He) and oxygen (O_2_) were used as carrier gases because we previously found that the addition of O_2_ to a He plasma improved the efficiency of cancer cell inhibition [10]. The emission spectra of the plasma according to the power used in this study (2 or 4 kV) is shown in Fig. 1B.

### Scanning electron microscopy (SEM)

For SEM, cells were grown on glass coverslips and fixed for 1 h at room temperature in 4% paraformaldehyde in phosphate-buffered saline (PBS) (pH 7.2). The coverslips were processed as described previously [17]. The cells were examined using a scanning electron microscope (S-4800; Hitachi, Tokyo, Japan) operating at 10 or 15 kV.

### Cytoskeleton staining

Cells were harvested and re-plated on fibronectin-coated coverslips. After 24 h, the cells were fixed for 20 min in 3.7% formaldehyde and re-hydrated in PBS with 0.1% Triton X-100. After blocking for 45 min with PBS containing 5% BSA, the slides were incubated with Texas-Red-conjugated phalloidin (Molecular Probes, Eugene, OR, USA) according to the manufacturer's instructions. Hoechst 33258 was added to counterstain the nuclei. The slides were analyzed using fluorescence microscopy (Carl Zeiss, Oberkochen, Germany).

### Pull-down assays (RhoA, Rac1, and Cdc42 GTPase activation assays)

To further determine the effect of NTP on morphological changes affecting cell migration, a RhoA/Rac1/Cdc42 Activation Assay Combo Biochem Kit (Cytoskeleton Inc., York, UK) was used according to the manufacturer's instructions with 600 µg of total protein. In brief, rhotekin-RBD effector domain affinity beads were used to bind active RhoA, and PAK-PBD effector domain affinity beads were used to bind active Cdc42 and Rac1. After incubation for 2 h, the proteins were eluted with Laemmli buffer and separated on 12% acrylamide/bis-acrylamide gels. Negative and positive controls were performed according to the manufacturer's instructions. To confirm the presence of GTPases in the cell protein extracts, 5% input controls were also run on the gels. The gels were transferred to nitrocellulose membranes, which were incubated overnight with antibodies against Cdc42, Rac1, and RhoA (included in the kit) with gentle agitation. The proteins were detected using an enhanced chemiluminescence Western blotting kit.

### Wound healing assays

For the cell migration assays, cells were plated in 12-well culture plates at a density of approximately 1×10^5^/well and grown to confluency. Wound healing assays were performed as described previously [10]. In brief, the monolayer was scratched with a sterile pipette tip, followed by extensive washing to remove cellular debris. The cells were then exposed to gas (He or O_2_ only), 2 or 4 kV of NTP, respectively, for 1 s. Wound healing ratios were documented by photography after 24 h since the doubling times for BHP10-3 and TPC1 were 30.0±1.2 h and 29.7±0.8 h, respectively. The doubling times were calculated from the cell growth curve over three days as follows: 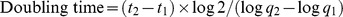 (t_2_: final time, t_1_: initial time, q_2_: final cell number, q_1_: initial cell number)

### Western blot analyses

Cells were lysed in lysis buffer containing 150 mM NaCl, 1.0% NP-40, 0.5% sodium deoxycholate, 0.1% SDS, 50 mM Tris (pH 8.0), and protease inhibitor cocktail (pH 7.4; Roche Applied Science, Vienna, Austria) as described previously [18]. The following antibodies were used for Western blotting: anti-focal adhesion kinase (FAK), -Src, -Paxillin, -extracellular signal-regulated kinase (ERK), -Akt, and -α-tubulin (1∶1000; Cell Signaling Technology, Danvers, MA, USA).

### Immunocytochemistry

Cells were cultured on microscope coverslips (Thermo Fisher Scientific, Rochester, NY, USA) and treated with gas (He+O_2_) only, 2, or 4 kV of NTP, respectively. After 24 h, the slides were washed with PBS, fixed for 20 min in 3.7% formaldehyde, and re-hydrated in PBS. After blocking for 45 min in BSA (in 5% PBS), the slides were incubated for 1 h with polyclonal rabbit anti-p-FAK and-paxillin antibodies (1∶50; Cell Signaling Technology), washed with PBS, and incubated for 45 min with Alexa 488-labeled goat anti-rabbit antibodies (1∶250; Molecular Probes). After rinsing in PBS, Hoechst 33258 was added for 15 min to counterstain the nuclei. The slides were washed with PBS and mounted with Vectashield (Vector Laboratories, Inc., Burlingame, CA, USA) then analyzed using fluorescence microscopy (Carl Zeiss).

### Invasion (Transwell) assays

Transwell (24-well) chambers (Costar, Cambridge, MA, USA) were used to evaluate cell invasion according to NTP treatment as described previously [10]. Initially, fibronectin (2 µg/filter) was dissolved in 100 µl of MEM and poured into the upper part of the polyethylene filter (pore size, 8 µm).The wells were coated overnight in a laminar flow hood.

Then, 10^5^ cells (in 100 µl of growth medium) were added to the top of the filter in the upper well. The chamber was incubated for 24 h in 5% CO_2_ at 37°C. Finally, attached cells in the lower section were stained with H&E, and counted using light microscopy.

### Reverse transcription-polymerase chain reaction (RT-PCR)

Total RNA was extracted from homogenized cells using TRIzol reagent (Gibco-BRL, Grand Island, NY, USA). ReverTraAce qPCR RT master mix (Toyobo Co. Ltd., Osaka, Japan) was used to reverse-transcribe RNA. Total RNA (1 µg) was mixed with 10 µg of the mixture. Reverse transcription was performed and cDNA was synthesized. The cDNA was added to a mixture of Quick Taq HS DyeMix (Toyobo Co. Ltd.) and specific primers, and amplified using a T100™ Thermal Cycler (Bio-Rad, Waltham, MA, USA). The matrix metalloproteinase (MMP)-2 and MMP-9 primer sequences were: MMP-2-F, 5′-ACC TGG ATG CCG TCG TGG AC-3′; MMP-2-R, 5′-TGT GGC AGC ACC AGG GCA GC-3′; MMP-9-F, 5′-GGG GAA GAT GCT GCT GTT CA-3′; and MMP-9-R, 5′-GGT CCC AGT GGG GAT TTA CA-3′.After denaturation for 3 min at 94°C, samples were amplified for 35 cycles of 30 s at 94°C, 60°C, and 72°C, with a 5-min extension at 72°C. The products were separated by electrophoresis in 1.5% agarose gels and detected using ultraviolet light (Bio-Rad, Hercules, CA, USA).

### Zymography

MMP-2/-9 activity was assayed using gelatin zymography as described previously [19]. BHP10-3 and TPC1 cells were treated with gas (He+O2) only, 2, or 4 kV of NTP for 1 s, and incubated for an additional 24 h. The supernatant (100 µl) from each sample was mixed with 1 µl of 100 mM 4-aminophenylmercuric acetate, and the samples were activated for 1 h at 37°C. Next, each sample was placed in sample buffer for 10 min and electrophoresed in polyacrylamide gels at 125 V for 120 min at 4°C using a Novex Xcell II system (Life Technologies, Carlsbad, CA, USA). The gels were incubated in renaturation buffer for 60 min at room temperature, followed by incubation for 18 h in 100 ml of developing buffer at 37°C with light shaking. The gels were then stained for 3 h with Coomassie brilliant blue. After decolorization in 400 ml of methanol, 100 ml of acetic acid, and 500 ml of distilled water, images were taken using an image analyzer.

### Urokinase-type plasminogen activator (uPA) assays

BHP10-3 and TPC1cells (3000 cells/well) were added to 96-well plates in complete medium containing 10% FBS. After overnight incubation, the cells were treated with gas (He+O_2_) only, 2, or 4 kV of NTP, respectively. The plates were then incubated for another 24 h. The cells were then processed as described previously [19]. Briefly, cells were washed with DMEM lacking phenol red and placed in 200 µl of reaction buffer containing 50% (v/v) of 0.05 U/ml plasminogen in DMEM (without phenol red), 40% (v/v) of 50 mM Tris-buffer (pH 8.2), and 10% (v/v) of 2.25 mM chromozyme PL in 100 mM glycine. The mixtures were incubated for 3 h at 37°C in 5% CO_2_. The absorbance at 405 nm was measured using an automated spectrophotometric plate reader.

### Statistical analyses

One-way analysis of variance (ANOVA) following post hoc Tukey's test were performed using SPSS 20.0 statistical software (SPSS, Chicago, IL, USA). Parameters of the data from three independent experiments are expressed as the mean ± S.D. A *P*<0.05 was considered statistically significant (*P<0.05; **P<0.01; ***P<0.001).

## Results

### NTP induces changes in cellular morphology in human thyroid papillary cancer cells

**Figure 2:**
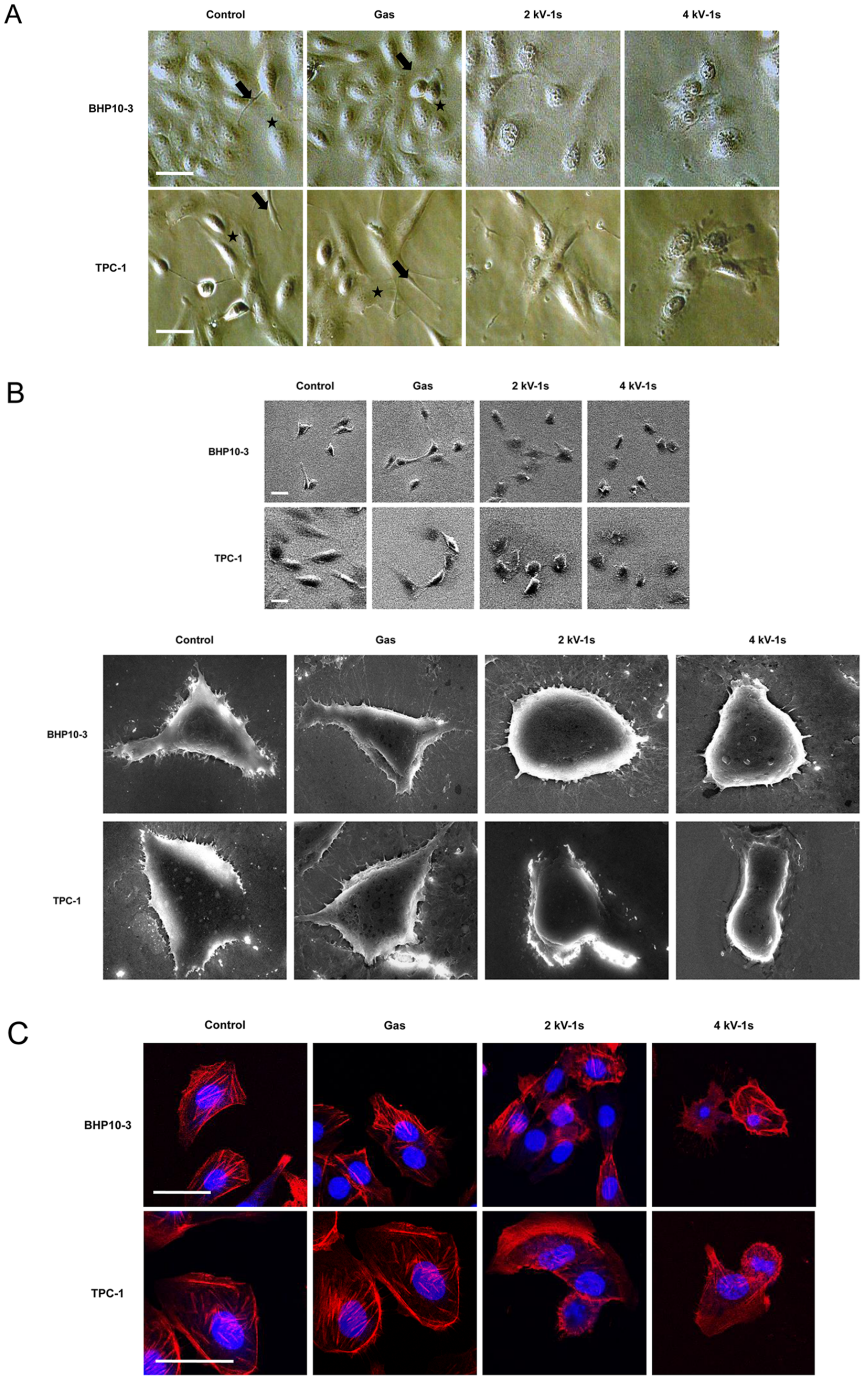
NTP affects cell morphology and cytoskeletal rearrangement in BHP10-3 and TPC1 cells. (A) After treatment with gas (He+O_2_) only, 2 or 4 kV of NTP for 1 s, respectively, cells were incubated for 24 h. The morphology of both cell lines was then examined by light microscopy. In the control and gas only-treated groups, the cells were flat and elongated, with lamellipodia (asterisk) and filopodia (arrow) that established cell-cell contacts laterally. In contrast, the NTP-treated cells showed morphologic changes characterized by a contracted cytoplasm, inhibited cell-to-cell contact, and abrogated cytoplasmic projections (lamellipodia and filopodia). (B) SEM images confirmed the effect of NTP on cell morphology. The cytoplasm of the NTP-treated cells showed shrinkage and a loss of horizontal polarization and cytoplasmic protrusions. Moreover, the surface of the cells treated with the plasma was rougher than that of the control and gas only-treated cells. (C) Immunofluorescence assays using Texas-Red-conjugated phalloidin were performed to visualize the cytoskeleton (F-actin); Hoechst 33258 was used to label cell nuclei. In the control and gas treated-cells, contractile actin bundles (stress fibers) and a diagonal actin filament meshwork were formed. However, in the NTP-treated groups, the actin filaments were distributed in the contracted cytoplasm destroying the architecture of the cytoskeleton, and no marked stress fiber formation was noted. Scale bar = 50 µm. Each figure was representative of three experiments with triplicates.

The surviving cells that adhered to the surface after NTP treatment exhibited a different morphology (Fig. 2A and B). Thyroid papillary cells normally grow in a flattened pattern, characterized by many cytoplasmic projections (lamellipodia and filopodia) [20]. After NTP treatment, the cells displayed a very different morphology that was characterized by a smaller and contracted appearance, and decreased cytoplasmic microspikes resulting in restricted cell spreading (Fig. 2A).

Scanning electron micrographs of both cell lines confirmed the above findings that control and gas only-treated cells showed mesenchymal-like features with many cytoplasmic projections. In contrast, the NTP-treated cells had more compact cell bodies and rough cell surfaces in which the protruding cytoplasmic processes were apparently decreased (Fig. 2B).

### NTP induces a dysregulated cytoskeletal architecture in human thyroid papillary cancer cells

We used immunocytochemistry against intracellular actin filaments with dye conjugated-phalloidin and found that the actin cytoskeletal structure of the plasma-treated cells was modified. In control and gas-treated cells, contractile actin bundles (stress fibers) and a diagonal actin filament meshwork were noted. However, the actin filaments were distributed in the contracted cytoplasm, thus destroying the architecture of the cytoskeleton, and no marked stress fiber formation was observed in NTP-treated cells (Fig. 2C).

### NTP inhibits the activity of Rho GTPases (RhoA, Rac1, and Cdc42)

**Figure 3:**
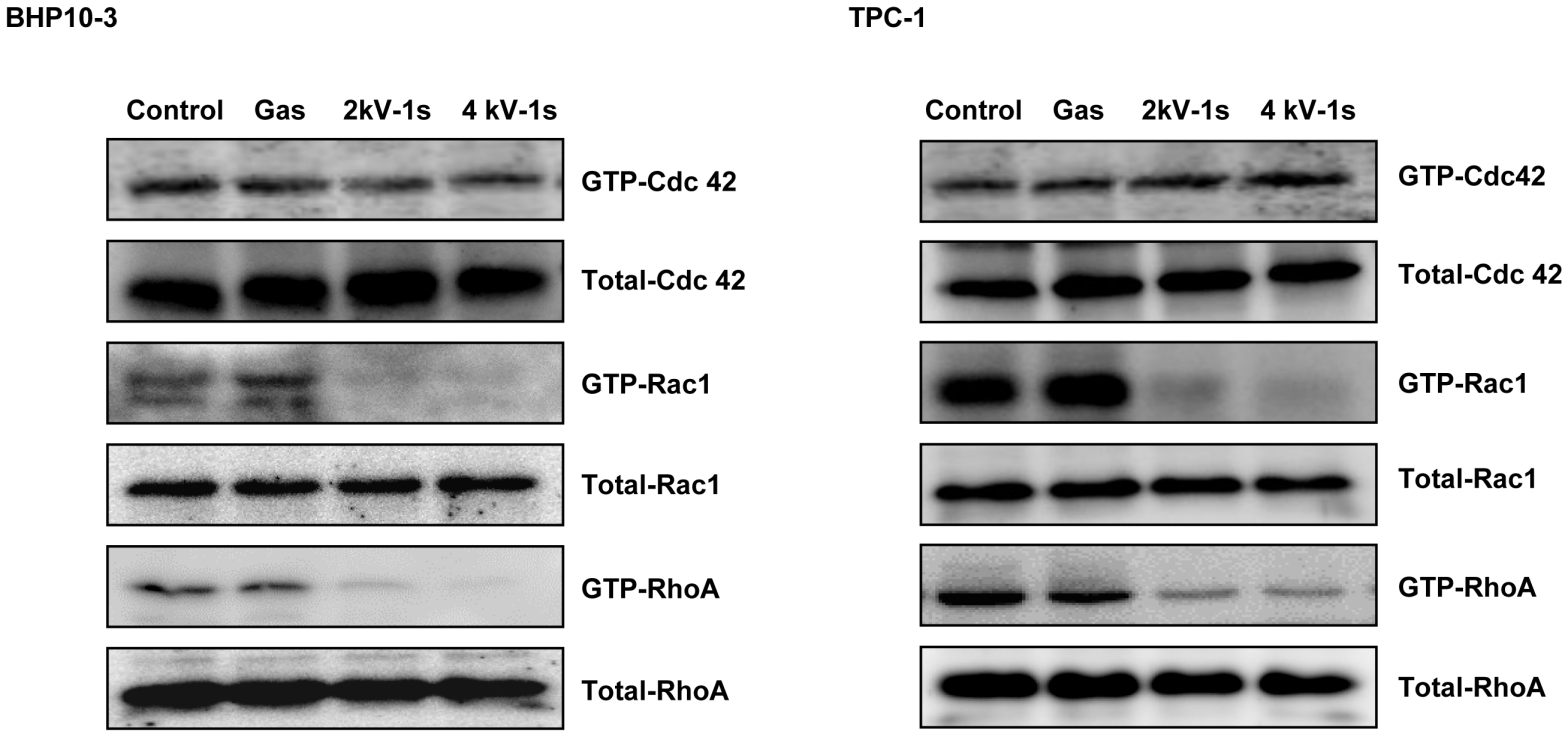
Pull-down assays. Rho family (Rho, Rac1, and Cdc42) activity was evaluated using pull-down detection kits after exposure to gas (He+O_2_) only, 2 or 4 kV of NTP for 1 s. NTP treatment of BHP10-3 and TPC1 cells significantly reduced the expression of GTP-RhoA and GTP-Rac1 (the active forms of these proteins). Each figure was representative of three experiments with triplicates.

The Rho family of GTPases is well known for its involvement in cellular functions such as cell polarity, lamellipodia or filopodia formation, and cell migration. Therefore, we investigated the effect of NTP on Rho family (RhoA, Rac1, and Cdc42) activity. As shown in Fig. 3, NTP treatment significantly decreased the active forms of RhoA and Rac1.

### NTP inhibits cell migration through the FAK/Src complex in human thyroid papillary cancer cells

**Figure 4:**
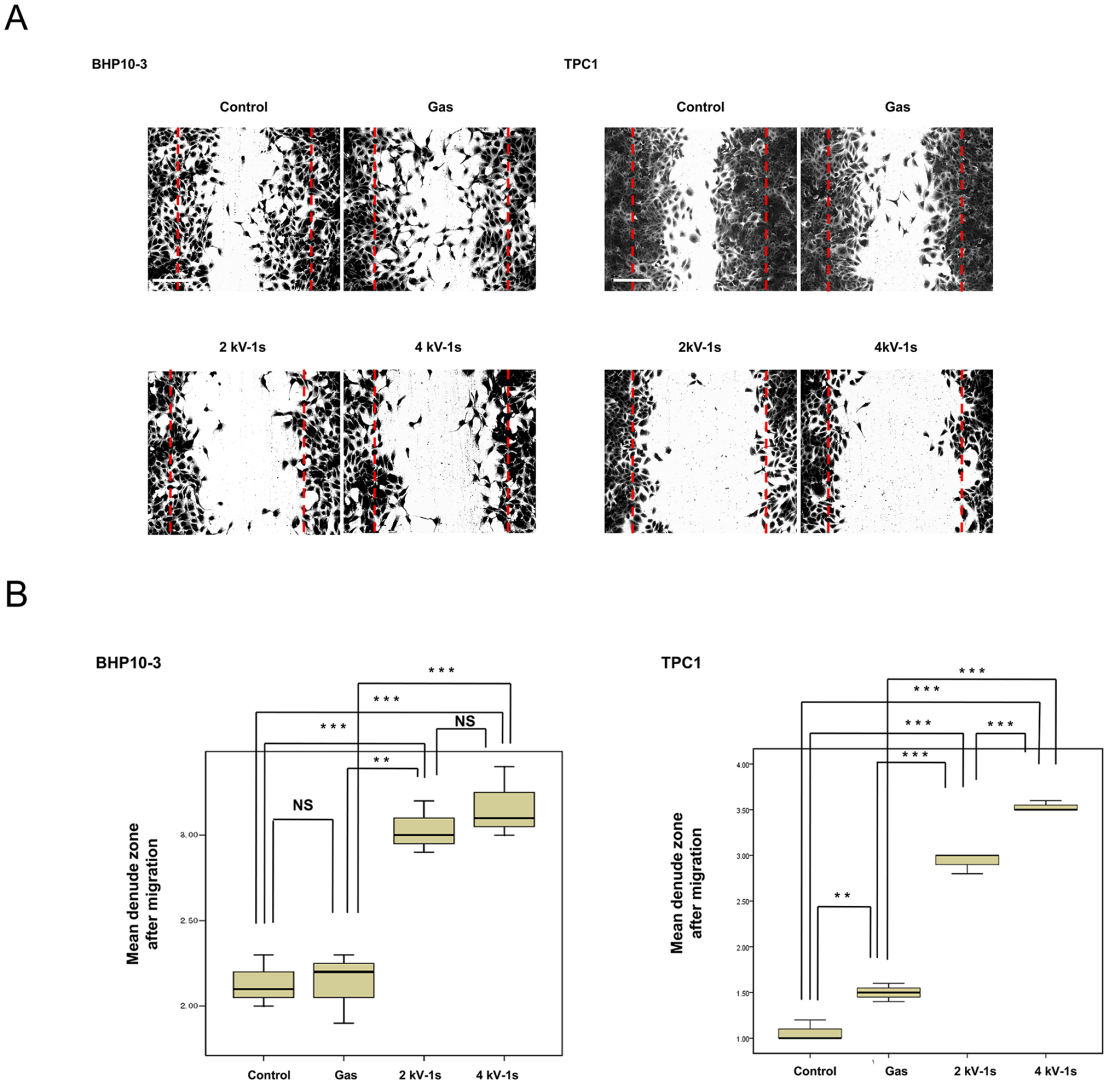
Wound healing assays. (A) BHP10-3 and TPC1 cells were plated in 12-well plates, grown to confluency, and the monolayer was wounded with a pipette tip. Wound healing was documented by photography after 24 h of incubation. Scale bar = 200 µm. (B) Quantification of cell migration. The NTP significantly inhibited the migration of BHP10-3 (*P*<0.001) and TPC1 (*P*<0.001) cells across the denuded zone. The data represent the mean ± S.D. of three independent experiments. NS, not significant; **P<0.01, ***P<0.001.

To investigate whether NTP reduces tumor cell migration, scratch wound closure assays were performed. As shown in Fig. 4A and B, our results demonstrate that plasma treatment significantly suppressed the migration of BHP10-3 (*P*<0.01) and TPC1 (*P*<0.001) cells across the denuded zone. The percent inhibition of cellular migration was 48.2 and 55.4% in BHP10-3 cells and 63.6 and 84.1% in TPC1 cells after 24 h of incubation with 2 and 4 kV of NTP, respectively, compared to control cells. Gas only treatment did not significantly affect cell migration in either line.

**Figure 5:**
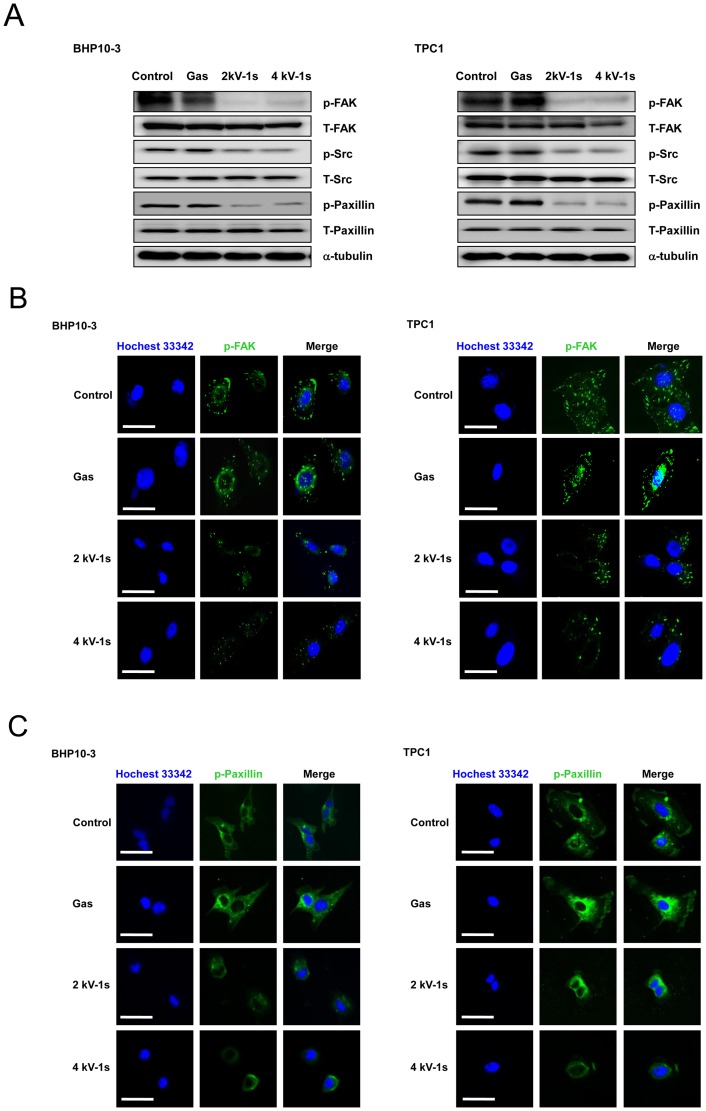
NTP treatment altered the expression of FAK, Src, and paxillin. (A) Western blotting for p-FAK, p-Src, and p-paxillin. Immunocytochemical assay for (B) p-FAK and (C) p-paxillin. In the control and gas treated-cells, FAK was localized in small adhesion structures at the cell periphery. After NTP treatment, FAK focal accumulation was significantly decreased in both cell lines. Moreover, p-paxillin staining was co-localized with p-FAK staining. NTP treatment also decreased p-paxillin expression. Scale bar = 50 µm. Each figure was representative of three experiments with triplicates.

To further evaluate the effect of NTP on cellular migration, we evaluated protein lysates for the phosphorylation of FAK, Src, and paxillin, which are known for their close association with cell migration, invasion, and cytoskeletal rearrangement via the FAK/Src kinase complex [21]. After NTP treatment, we observed the inhibition of FAK phosphorylation and a consistent reduction in the phosphorylation of Src and paxillin, which are downstream of FAK (Fig. 5A).

We also analyzed the intracellular distribution of phosphorylated (p-)FAK and phosphorylated (p-)paxillin. In control and gas only-treated cells, p-FAK accumulated in well-defined zones, including the cell periphery, whereas after NTP treatment, p-FAK staining was significantly decreased (Fig. 5B). Co-localization of the signals for p-paxillin and p-FAK was noted (Figs. 5B and C).

### NTP inhibits cell invasion by decreasing MMP-2/-9 and uPA activity, and Akt and ERK signaling in human thyroid papillary cancer cells

**Figure 6:**
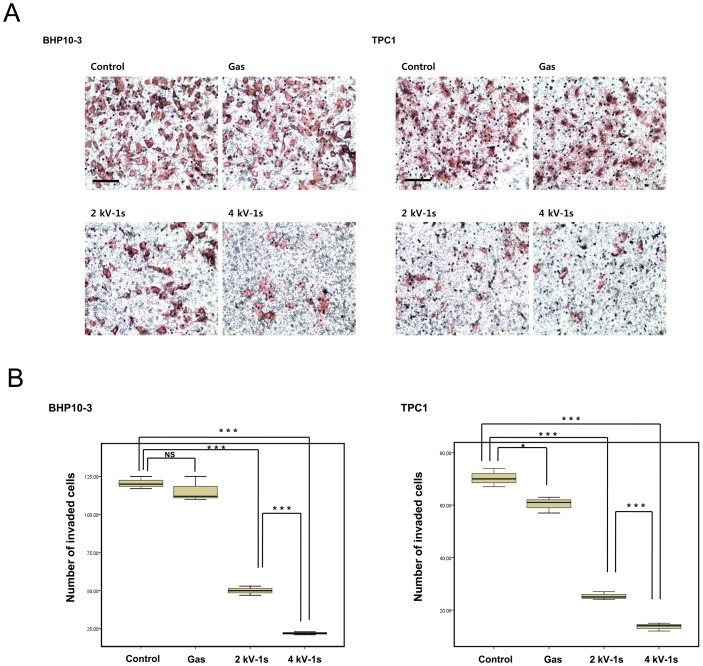
Invasion assays. (A) BHP10-3 and TPC1 cells were seeded on filters (pore size, 8 µm) coated with Matrigel in the upper compartment and exposed to He+O_2_ gas only, 2 or 4 kV of NTP for 1 s. After 24 h, the cells in the pores or cells attached to undersurface of the membrane were counted, and those cells attached to the lower section were stained with H&E and counted using light microscopy (200×). Scale bar = 100 µm. (B) Quantification of the invasion assay data. NTP treatment significantly reduced the number of BHP10-3 (*P*<0.001) and TPC1 (*P*<0.001) cells that penetrated the membrane. The data represent the mean ± S.D. of three independent experiments. *P<0.05, **P<0.01, ***P<0.001.

Previous studies have demonstrated that FAK regulates cell invasion as well as migration, survival, and metastasis in a variety of cell types, including thyroid cancer [22], [23]. To investigate whether NTP reduces tumor invasion, invasion assays were performed using Transwell chambers and Matrigel. Tumor invasion requires degradation of the basement membrane and ECM, cytoplasmic extension, and cell migration. Transwell assays mimic this environment for tumor invasion. The attached cells in the lower section passed through the filter of the chamber, indicating invasive cells. As shown in Fig. 6A, H&E-stained cells were present on the undersurface of the membrane 24 h after incubation. However, plasma treatment significantly inhibited the number of invading cells compared to untreated cells (*P*<0.001) (Fig. 6B).

**Figure 7:**
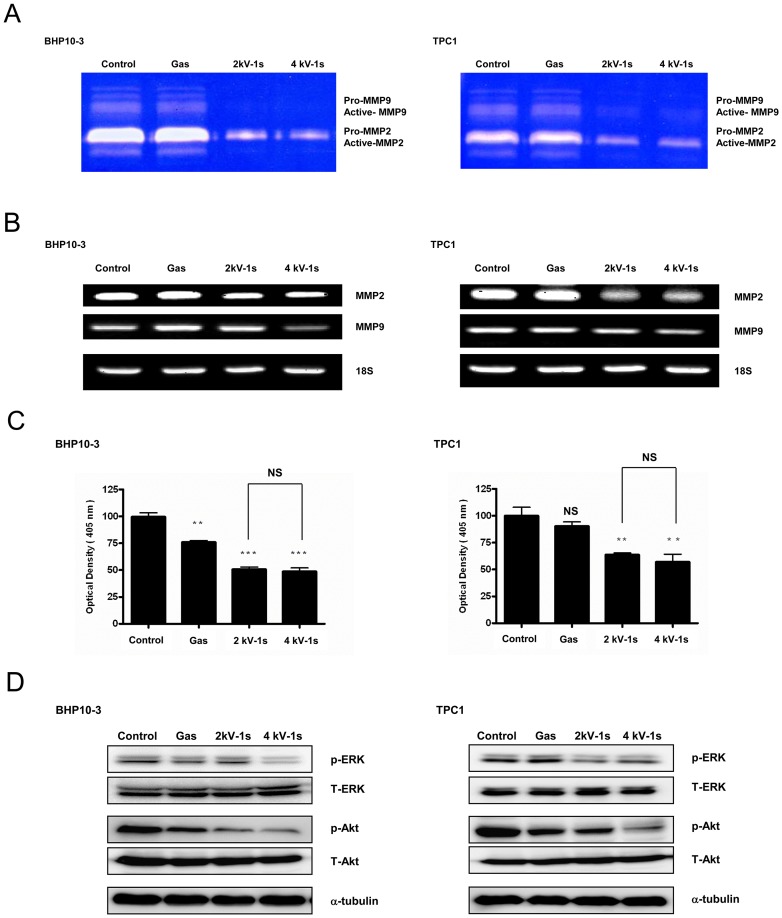
NTP decreases MMP-2/-9 and uPA activity in BHP10-3 and TPC1 cells. (A) Gelatin zymography for MMP-2/-9. NTP attenuated MMP-2/-9 enzymatic activity in both cell lines. (B) RT-PCR for MMP-2/-9. The mRNA levels of MMP-2/-9 decreased after NTP treatment in both cells. Each figure was representative of three experiments with triplicates. (C) uPA assays. NTP treatment significantly decreased uPA activity. The data represent the mean ± S.D. of three independent experiments. NS, not significant, **P<0.01, ***P<0.001. (D) Western blotting. ERK and Akt expression was significantly decreased after NTP treatment in an intensity-dependent manner. Each figure was representative of three experiments with triplicates.

To understand the mechanism by which NTP impacts tumor invasion *in vitro*, gelatin zymography for MMP-2/-9 activity were performed. Our results revealed a noticeable reduction in MMP-2/-9 activity when BHP10-3 and TPC1 cells were treated with NTP for 1 s (Fig. 7A). To further identify the influence of NTP on MMPs, RT-PCR was utilized to evaluate the mRNA expression of MMP-2/-9. As shown in Fig. 7B, NTP treatment markedly inhibited MMP-2/-9 mRNA expression. These findings suggest that the NTP inhibited cell invasion by decreasing the transcription of MMP-2/-9 and inhibiting the activity of the existing enzyme. The NTP also inhibited uPA activity as demonstrated by uPA assays (Fig. 7C).

Finally, the protein expression of Akt and ERK was examined using Western blotting. NTP treatment inhibited Akt and ERK expression in both cell lines compared to the control or gas only group (Fig. 7D).

## Discussion

Our current data indicate that NTP exerted an anti-tumor effect by inhibiting cancer cell migration and invasion. NTP is a promising and emerging modality in cancer research, and we previously reported that the anti-cancer effects of NTP are mediated through a growth arrest and cell death [10], [11]. However, the first point of contact between an NTP and cells is the cell membrane, which contains various proteins performing dynamic functions. Therefore, in this study we focused on changes in cell appearance that occurred on the cell surface after NTP treatment. Our data show that the plasma-treated thyroid cancer cells lost their flat, spindle-shaped horizontal polarization and had decreased cytosolic projections, which are important for cellular mobility and environmental sensing, whereas the cells did not show significant apoptosis by NTP treatment (Supplementary fig. S1). These actin-based cellular protrusions, called lamellipodia (actin filament meshwork) and filopodia (radially-oriented actin filaments), are controlled by Rho family small GTP-binding proteins (Rho-GTPases; RhoA, Rac1, and Cdc42) [24]. These highly regulated proteins also control the mesenchymal-like polarization of cells and cytoskeletal rearrangements, which are the first step in migration [24]. We confirmed the induction of morphologic changes by the NTP through staining of the cytoskeleton, which revealed actin fiber rearrangement. Taken together, our migration and pull-down assay data indicate that the plasma effectively inhibited BHP10-3 and TPC1 cell migration, thus relating cell morphologic changes to cytoskeletal rearrangement.

FAK is a non-receptor tyrosine kinase that localizes at focal adhesions. FAK mRNA and protein expression is increased in the vast majority of invasive and metastatic tumors, including human thyroid cancer [25]. However, FAK is barely detectable in normal tissues or non-invasive tumors [25]–[28]. Moreover, it is one of the most prominently phosphorylated proteins in thyroid papillary cancer cells [29]. Studies have shown that FAK promotes cell migration through complex formation with Src, and subsequent phosphorylation of the cytoskeletal adaptor molecule paxillin by the FAK/Src complex [30]. In addition, paxillin is associated with the Cdc42/Rac target effector, which may link Cdc42 and Rac to other kinases and downstream targets [31], [32]. FAK is also known to regulate cell migration by modulating the assembly and disassembly of the actin cytoskeleton through its effects on the Rho subfamily of small GTPases [33], [34]. The results of this study are in agreement with these earlier studies because we found that NTP significantly inhibited cell migration by suppressing expression of the FAK/Src complex and paxillin signaling, which are related to cytoskeletal regulation.

In addition to its well-characterized role in cell migration, FAK signaling is related to tumor invasion by a number of mechanisms [35], [36]. For example, the MMP/uPA system plays a major role in ECM degradation and is crucial for facilitating tumor migration and invasion during metastasis [37]. Therefore, we investigated the effect of NTP on tumor cell invasion and the FAK and MMP/uPA system. The relationship between FAK and the MMP-2/-9 pathway has been previously investigated [22], [38]. FAK inhibition has been shown to reduce MMP-9 secretion in carcinoma cells [39]. In addition, uPA binding to urokinase plasminogen activator receptor activates the conversion of plasminogen to plasmin. Activated plasmin mediates the conversion of pro-MMPs to MMPs. Therefore, MMP/uPA inhibition is closely related to the decreased invasiveness of cancer cells. In this study, the results with zymography and RT-PCR indicate that NTP treatment induced decrease in MMP-2/-9 expressions and their activities, also. These data were in accordance with previous published studies in which the expressions of pro-MMP-2/-9 and their enzyme activities are closely correlated with malignant and/or metastatic phenotype of cancer [40]–[42]. Taken together, our current results show that NTP effectively decreases cancer cell invasiveness via the MMP-2/-9/uPA system, which is associated with Akt and ERK signalings. Previous data revealed that the MMP/uPA system is regulated through the PI3K-Akt and/or ERK/p38 signalings [22], [43], [44].

We also examined the effect of NTP in the normal thyroid cell line (Nthy-ori 3-1). NTP treatment did not induce significant apoptotic cell death on normal thyroid cells (Supplementary figs. S2 and S3). Moreover, in contrast to the tumor cells, normal thyroid cells did not show significant morphologic changes (Supplementary fig. S4) and reduction in cell migration after NTP treatment (Supplementary fig. S5) suggesting differential sensitivity to NTP treatment between cancer cells and normal thyroid cells. These results are in accordance with previous studies, which reported the selective anticancer effect of plasma treatment [5], [45]. Although, further studies should be performed to fully elucidate the underlying mechanism of preferential anticancer effect, these results suggest the NTP as an efficacious modality in thyroid cancer therapy.

In summary, this study demonstrates that NTP inhibited cell migration and invasion in BHP10-3 and TPC1 cells. Mechanistically, we demonstrated that the anti-cancer activity of NTP involves cytoskeletal rearrangements, resulting in changes in cellular morphology, as well as inhibition of the MMP-2/-9/uPA system and Akt/ERK signalings. Moreover, both signal pathways are related to NTP-mediated FAK inhibition. Since active migration and invasion of tumor cells are prerequisites for metastasis of cancer [46], our results suggest that NTP could open up new avenue for further investigations on abrogating tumor invasion and metastasis, and these also provide a novel mechanistic explanation of the anti-cancer effects of NTP.

## Supporting Information

Figure S1 **NTP did not induced significant apoptotic cell death in BHP10-3 and TPC1 cells.** Cells were treated with gas (He+O_2_) only or plasma jets at 2 kV and 4 kV for 1 s and then incubated for 24 h. (A) After harvest and then washing with phosphate-buffered saline (PBS), the cells were stained with annexin V/propidium iodide (PI). (B) Quantification of the annexin V/PI assay. Early and late apoptosis were quantified from three independent three experiments. NTP treatment did not induced significant apoptosis in both BHP10-3 and TPC1 cells. NS, not significant.
(TIF)

Figure S2 **NTP had little effect on the viability of Nthy-ori 3-1 cells.** After treatment with gas (He+O_2_) only, 2 or 4 kV of NTP for 1 s, respectively, cells were incubated for 24 h. Then, the cell viability was estimated using the 3-(4,5-dimethylthiazol-2-yl)-2,5-diphenyltetrazolium bromide (MTT) assay. The data represent the mean ± S.D. of three independent experiments. NS, not significant.
(TIF)

Figure S3 **NTP did not induced significant apoptotic cell death in Nthy-ori 3-1 cells.** Cells were treated with gas (He+O_2_) only or plasma jets at 2 kV and 4 kV for 1 s and then incubated for 24 h. (A) The cells were harvested and washed with phosphate-buffered saline (PBS), and stained with annexin V/propidium iodide (PI). (B) Quantification of the annexin V/PI assay. Early and late apoptosis were quantified from three independent three experiments. NTP treatment did not induced significant apoptosis in Nthy-ori 3-1 cells. NS, not significant.
(TIF)

Figure S4 **NTP had no effect on cell morphology and cytoskeletal arrangement in Nthy-ori 3-1 cells.** After treatment with gas (He+O_2_) only, 2 or 4 kV of NTP for 1 s, respectively, cells were incubated for 24 h. The morphology of both cell lines was then examined by light microscopy. The cells of every group were flat and elongated, with lamellipodia (asterisk) and filopodia (arrow). Scale bar = 50 µm. Each figure was representative of three experiments with triplicates.
(TIF)

Figure S5 **Wound healing assay of normal thyroid cell.** (A) Nthy-ori 3-1 cells were plated in a 12-well plate and grown to confluency, and the monolayer was wounded with a pipette tip. To evaluate the effect of NTP on both migratory activities, the cells exposed to 2 kV and 4 kV of NTP for 1 sec in the presence of media. Wound healing was documented by photography after 24 h incubation (magnification: ×100). Scale bar = 200 µm (B) Quantification of cell migration assay from three independent three experiments. NS, not significant.
(TIF)
