# Supplementary figures and images for: Non-Thermal Atmospheric Pressure Plasma Inhibits Thyroid Papillary Cancer Cell Invasion via Cytoskeletal Modulation, Altered MMP-2/-9/uPA Activity
Source: PLoS One. 2014 Mar 25;9(3):e92198. doi: 10.1371/journal.pone.0092198 (PMC3965425; doi:10.1371/journal.pone.0092198)

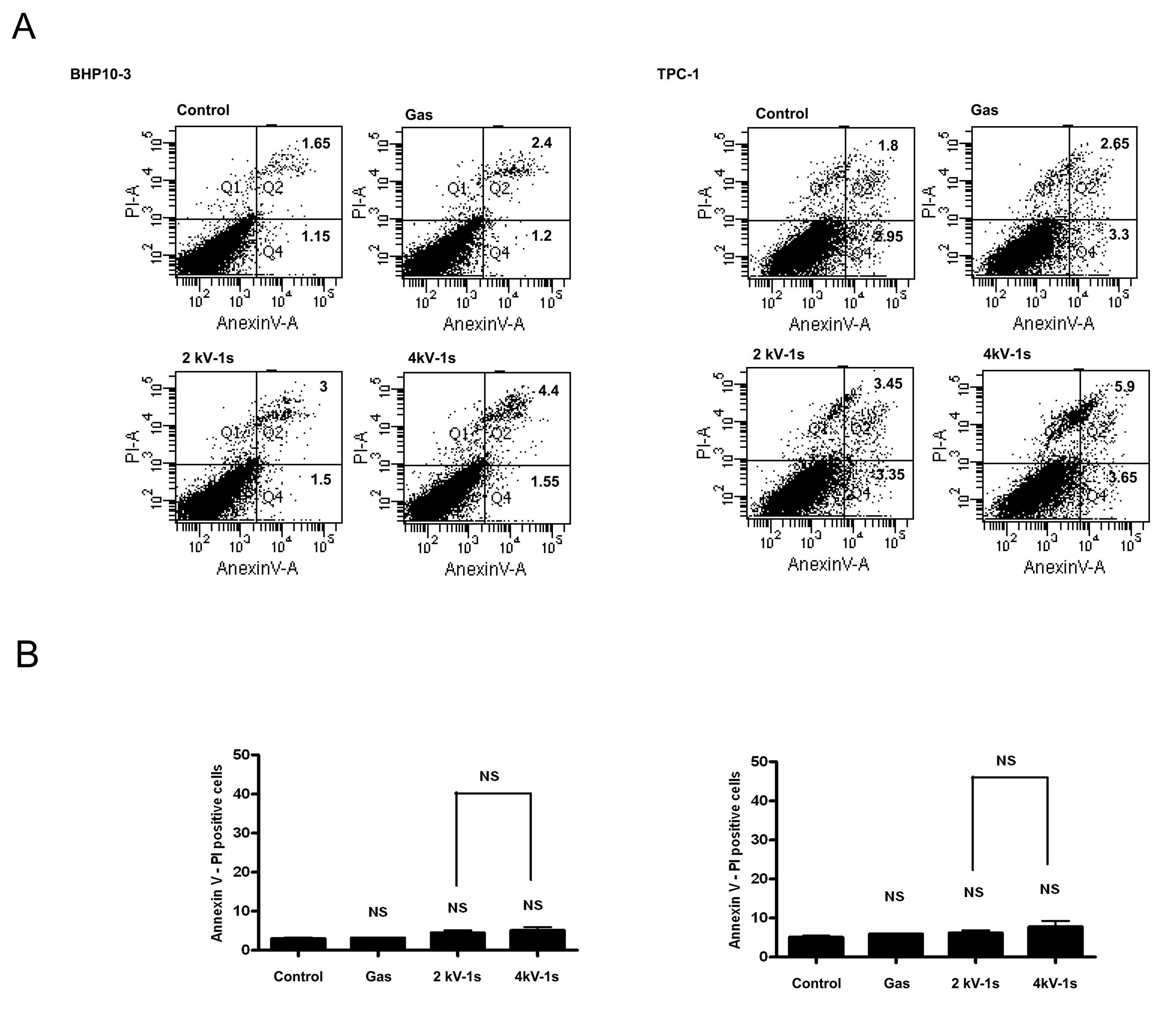

Supplement: Figure S1 — NTP did not induced significant apoptotic cell death in BHP10-3 and TPC1 cells. Cells were treated with gas (He+O2) only or plasma jets at 2 kV and 4 kV for 1 s and then incubated for 24 h. (A) After harvest and then washing with phosphate-buffered saline (PBS), the cells were stained with annexin V/propidium iodide (PI). (B) Quantification of the annexin V/PI assay. Early and late apoptosis were quantified from three independent three experiments. NTP treatment did not induced significant apoptosis in both BHP10-3 and TPC1 cells. NS, not significant. (TIF) [file pone.0092198.s001.tif]

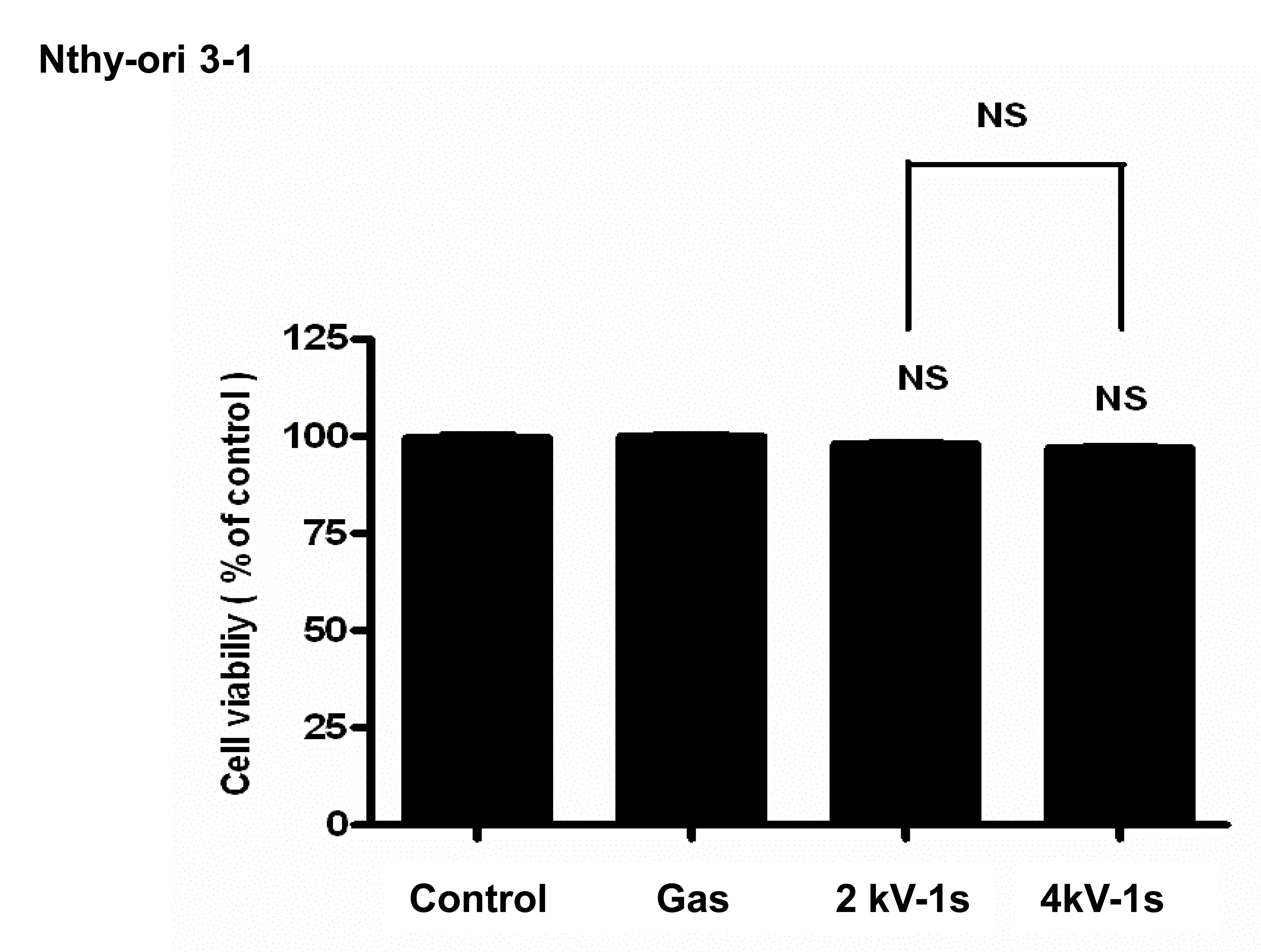

Supplement: Figure S2 — NTP had little effect on the viability of Nthy-ori 3-1 cells. After treatment with gas (He+O2) only, 2 or 4 kV of NTP for 1 s, respectively, cells were incubated for 24 h. Then, the cell viability was estimated using the 3-(4,5-dimethylthiazol-2-yl)-2,5-diphenyltetrazolium bromide (MTT) assay. The data represent the mean ± S.D. of three independent experiments. NS, not significant. (TIF) [file pone.0092198.s002.tif]

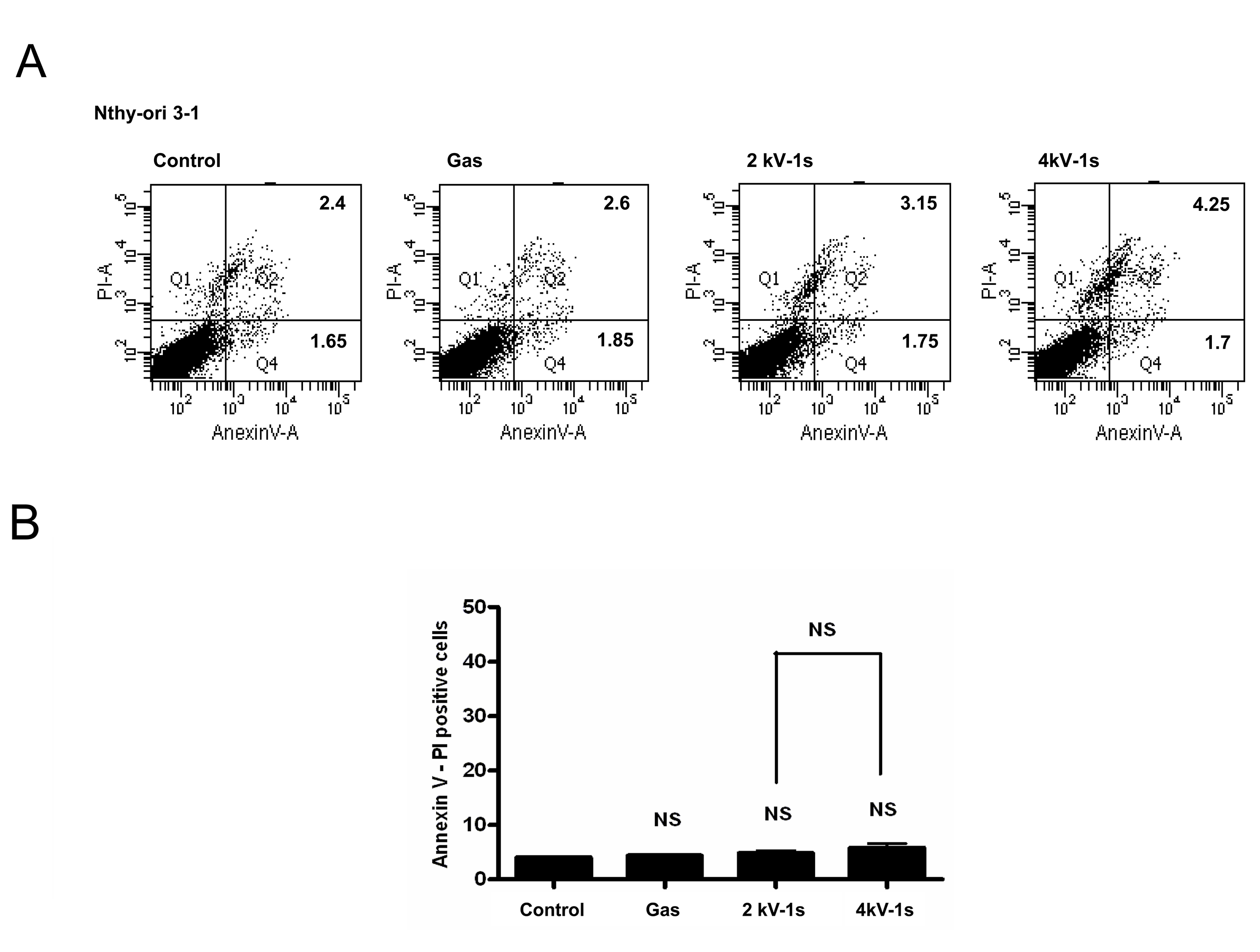

Supplement: Figure S3 — NTP did not induced significant apoptotic cell death in Nthy-ori 3-1 cells. Cells were treated with gas (He+O2) only or plasma jets at 2 kV and 4 kV for 1 s and then incubated for 24 h. (A) The cells were harvested and washed with phosphate-buffered saline (PBS), and stained with annexin V/propidium iodide (PI). (B) Quantification of the annexin V/PI assay. Early and late apoptosis were quantified from three independent three experiments. NTP treatment did not induced significant apoptosis in Nthy-ori 3-1 cells. NS, not significant. (TIF) [file pone.0092198.s003.tif]

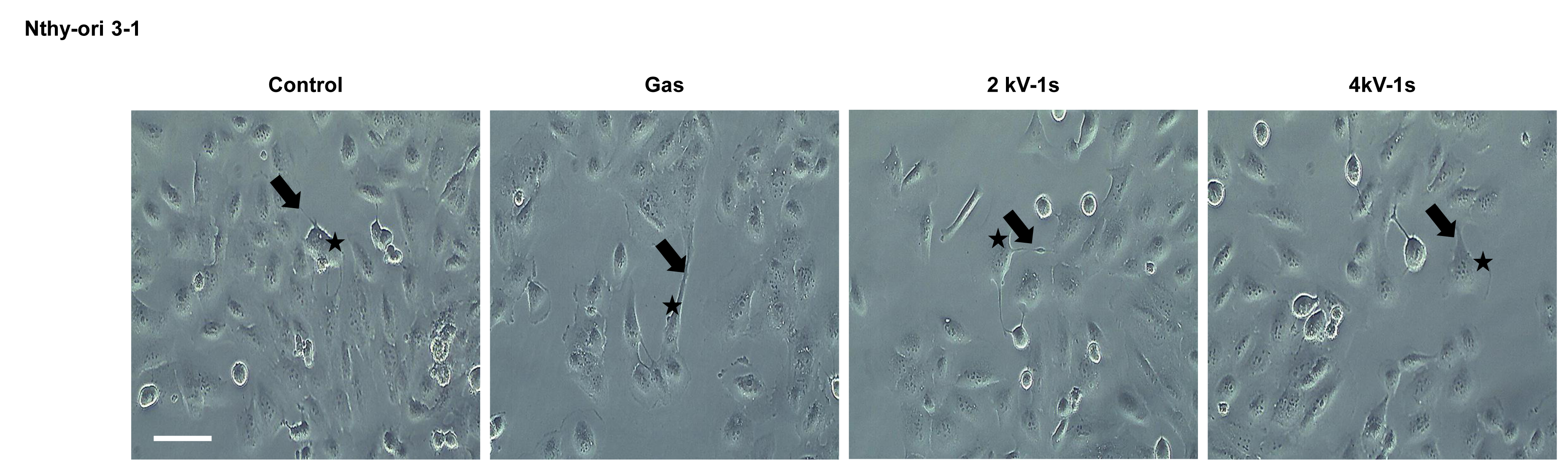

Supplement: Figure S4 — NTP had no effect on cell morphology and cytoskeletal arrangement in Nthy-ori 3-1 cells. After treatment with gas (He+O2) only, 2 or 4 kV of NTP for 1 s, respectively, cells were incubated for 24 h. The morphology of both cell lines was then examined by light microscopy. The cells of every group were flat and elongated, with lamellipodia (asterisk) and filopodia (arrow). Scale bar = 50 µm. Each figure was representative of three experiments with triplicates. (TIF) [file pone.0092198.s004.tif]

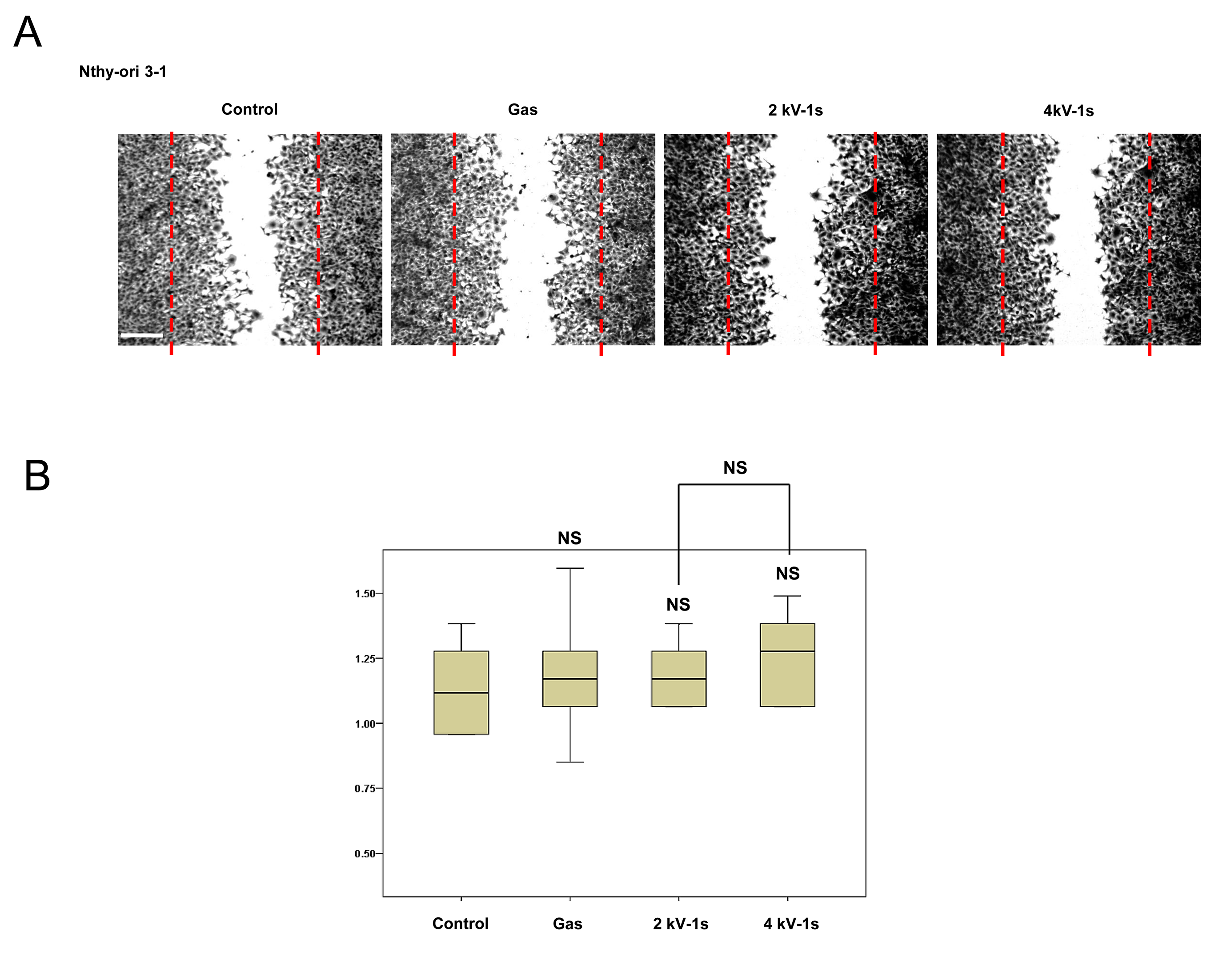

Supplement: Figure S5 — Wound healing assay of normal thyroid cell. (A) Nthy-ori 3-1 cells were plated in a 12-well plate and grown to confluency, and the monolayer was wounded with a pipette tip. To evaluate the effect of NTP on both migratory activities, the cells exposed to 2 kV and 4 kV of NTP for 1 sec in the presence of media. Wound healing was documented by photography after 24 h incubation (magnification: ×100). Scale bar = 200 µm (B) Quantification of cell migration assay from three independent three experiments. NS, not significant. (TIF) [file pone.0092198.s005.tif]
